# Risk factors associated with secondary displacement in fractures of the humeral greater tuberosity

**DOI:** 10.3389/fsurg.2025.1474983

**Published:** 2025-04-24

**Authors:** Qing-Quan Chen, Han-Lin Chen, Hong-Shen Wang, Xiao-Li Huang, Jin-Shui Chen, Xiu Yang

**Affiliations:** ^1^ Spinal Ward, Fuzong Clinical Medical College of Fujian Medical University, FuZhou, China; ^2^ Spinal Ward, The 900th Hospital of PLA Joint Logistic Support Force, FuZhou, China

**Keywords:** greater tuberosity of humerus, fracture, secondary displacement, risk factors, fractures of the proximal humerus

## Abstract

**Background:**

The incidence of secondary displacement in fractures of the greater tuberosity of the humerus remains high, irrespective of whether conservative or surgical treatment is administered. However, the specific risk factors contributing to secondary displacement of the greater tuberosity of the humerus have not been previously reported. The primary objective of this study was to analyze the risk factors associated with secondary displacement of the greater tuberosity of the humerus and to summarize corresponding guidelines for clinical diagnosis and treatment.

**Methods:**

A retrospective analysis was conducted on patients with fractures of the greater tuberosity of the humerus who received treatment at the same trauma center between January 2018 and December 2022. The following variables were recorded for each patient: age, gender, injured limb (left/right), whether the fracture was comminuted, bone density, fracture displacement, shoulder joint dislocation, treatment plan, and treatment outcomes, including the success rate of reduction and the time of secondary displacement. The patients were categorized into two groups based on the absence or presence of secondary displacement. For statistical analysis, the Mann–Whitney *U* test and logistic regression analysis were employed. The significance level was set at *P* < 0.05.

**Results:**

Among the 177 patients enrolled in this study, 144 (81.36%) did not exhibit secondary displacement, while 33 (18.64%) did present with such displacement. Significant statistical differences were observed between the two groups in mean age, fracture type, bone mineral density, shoulder dislocation, and reduction quality of fracture, indicating a statistically significant association (*P* < 0.05). However, no significant difference was found in gender, Left/right limb**s**, displacement of fracture, and treatment method (*P* > 0.05). Logistic regression analysis revealed that comminuted fractures, osteoporosis, shoulder dislocation and poor reduction independently contributed to an increased risk of secondary displacement of the greater tuberosity of humerus.

**Conclusions:**

Comminuted fracture, osteoporosis, shoulder dislocation, and poor reduction have been identified as independent risk factors for secondary displacement. In the course of clinical diagnosis and treatment, it is imperative to consider the potential adverse prognosis that may be associated with these conditions.

## 1 Introduction

Fractures of the greater tuberosity of the humerus, which often result from high-energy trauma with or without associated dislocation, account for 20% of proximal humeral fractures. Between 5% and 57% of greater tuberosity fractures are associated with anterior glenohumeral dislocation, whereas fractures of the greater tuberosity constitute 15%–30% of cases involving anterior glenohumeral dislocation (1, 2). Currently, it is widely acknowledged that surgical intervention is recommended for individuals with fracture displacement of ≥5 mm or fracture displacement of ≥3 mm accompanied by high functional demands. However, there is presently no universally accepted surgical protocol established as the gold standard in clinical practice (3). The primary objectives of treating humeral greater tuberosity fractures include achieving anatomical reduction, promoting stable osseointegration, and facilitating early rehabilitation. However, achieving these goals in every case can be challenging. Open reduction with titanium plate internal fixation, hollow screw and anchor internal fixation are the most commonly employed treatment methods for humerus greater tuberosity fractures. With the advancement of minimally invasive approaches, arthroscopy is increasingly being utilized for the management of greater tuberosity fractures (4–7). However, despite the continuous advancement of treatment techniques, the incidence of complications associated with greater tuberosity fractures remains unchanged. Common complications include implant failure, recurrent displacement of the greater tuberosity fragment, and acromial impingement (8, 9). The findings of various studies have substantiated a direct correlation between the extent of upward displacement of the greater tuberosity of the humerus and the force exerted by the supraspinatus muscle. Consequently, increased displacement necessitates elevated tension within the supraspinatus muscle to maintain normal shoulder joint functionality (10).

Currently, there remains a significant incidence of secondary displacement of greater tuberosity bone fragments following conservative or surgical treatment. Surgeons have increasingly focused on employing advanced surgical techniques and internal fixation materials to achieve improved reduction outcomes in cases of greater tuberosity fractures. However, the risk factors contributing to secondary displacement of the greater tuberosity have not been extensively reported. We postulate that potential risk factors associated with secondary displacement of a greater tuberosity fracture encompass age, gender, injured limb (left/right), whether the fracture was comminuted, bone density, fracture displacement, shoulder joint dislocation, treatment plan, etc. To substantiate this hypothesis, the present study retrospectively examined patients admitted to the same trauma center between 2018 and 2022 who presented with greater tuberosity fractures and analyzed the aforementioned risk factors for their association with secondary displacement. Furthermore, we aim to derive comprehensive guidelines for clinical diagnosis and treatment based on our findings.

## 2 Methods

### 2.1 Study design and setting

This retrospective chart review of electronic medical records for patients with simple fractures of the humeral greater tuberosity was conducted as an institutional review board-approved, single-institutional study. Prior to initiation, ethical approval was obtained from the Biomedical Ethics Committee of the 900th Hospital of the Joint Logistics Support Force (Ethics Review No. 2024-008). Informed consent was obtained from all participants included in the study, and all procedures adhered to relevant guidelines and regulations.

### 2.2 Participants

**Figure 1:**
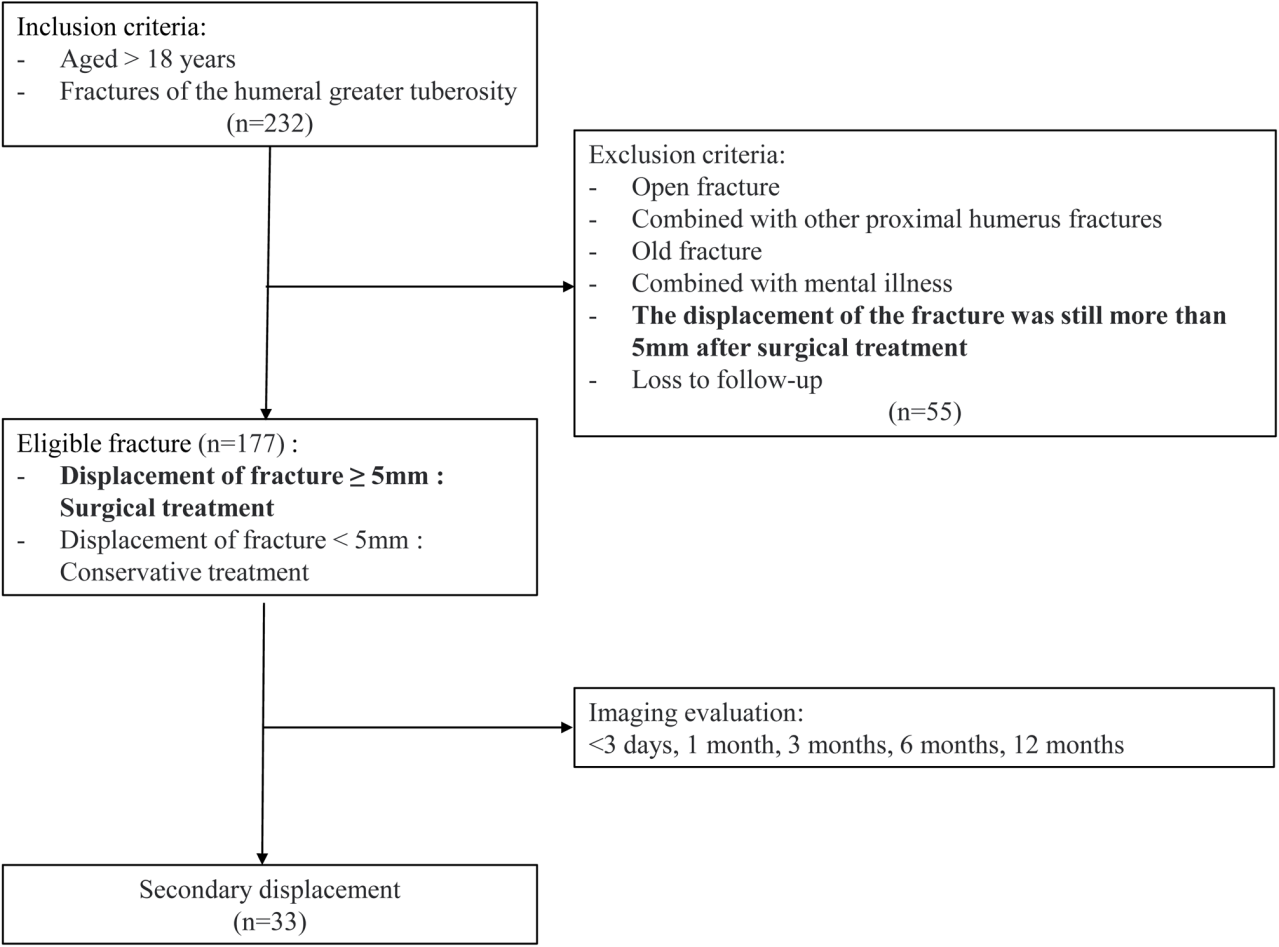
Flowchart of inclusion and exclusion criteria.

We performed a retrospective analysis on patients with proximal humerus fractures who had received treatment at our trauma center between January 2018 and December 2022. The inclusion criteria encompassed patients aged 18 years or older with fractures of the humeral greater tuberosity, who sought medical attention within 24 h of injury and underwent regular follow-up until complete fracture healing. Fractures were classified as either simple (non-comminuted) or comminuted (multiple fragments) based on radiographic and CT imaging. This classification was used to evaluate the impact of fracture complexity on treatment outcomes, particularly secondary displacement. Exclusion criteria were as follows: (1) open fracture; (2) combined with other proximal humerus fractures; (3) old fracture (defined as fractures presenting >3 weeks post-injury with radiographic evidence of early healing, malunion, or lack of reducibility due to callus formation); (4) combined with mental illness; (5) fracture displacement greater than 5 mm after surgical treatment; (6) loss to follow-up. The schematic diagram of inclusion and exclusion criteria is shown in Figure 1. Detailed information of the patients was recorded, including age, gender, injured limb (left/right), whether the fracture was comminuted, bone density, fracture displacement, shoulder joint dislocation, treatment plan, and treatment outcomes, including the success rate of reduction and the time of secondary displacement.

### 2.3 Perioperative management and follow-up

**Figure 2:**
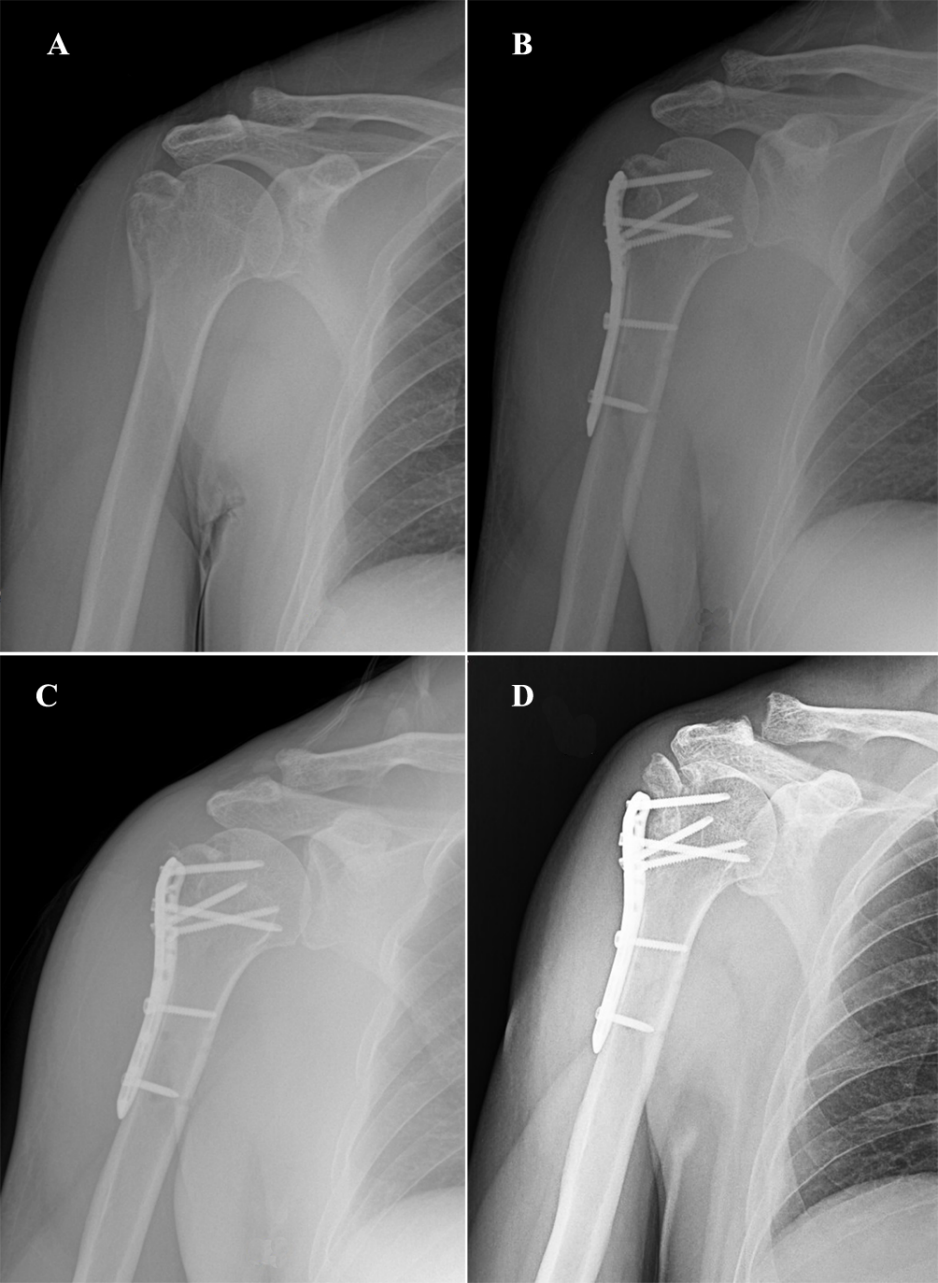
A 56-year-old male patient presenting with a comminuted fracture of the greater tuberosity of the humerus. **(A)** X-ray image of the shoulder joint obtained within 24 h post-injury; **(B)** x-ray image of the shoulder joint captured 1 day post-surgery; **(C)** x-ray image of the shoulder joint taken 1 month post-surgery; **(D)** x-ray image of the shoulder joint obtained 3 months post-surgery.

All patients underwent immediate shoulder joint x-ray and three-dimensional CT examination following injury onset for initial assessment. Follow-up assessments were conducted on the 3rd day, and at 1, 3, 6, 12 months post-treatment. These assessments included x-ray examinations of the shoulder joint to evaluate fracture healing and detect any secondary displacement of the greater tuberosity of the humerus. In cases accompanied by shoulder joint dislocation, x-ray and CT scans should be performed immediately after manual reduction to assess the effect of joint reduction and any potential displacement of the greater tuberosity of the humerus. Conservative management was pursued for patients presenting with less than 5 mm of displacement in the greater tuberosity fracture, whereas open reduction and internal fixation were employed for those exhibiting displacement exceeding 5 mm. Within 3 days after the operation, x-ray and CT scans of the shoulder joint were performed to evaluate the quality of reduction of the greater tuberosity of the humerus. All patients underwent dual-energy x-ray absorptiometry (DXA) to assess bone mineral density (BMD) at the lumbar spine during their initial hospitalization. In this study, regardless of conservative treatment or surgical treatment, the fracture of the tuberosity of the humerus experienced redisplacement during follow-up evaluation, exceeding 5 mm again, which we refer to as secondary displacement. Figure 2 illustrates a case demonstrating such secondary displacement after surgery.

### 2.4 Conservative management

#### 2.4.1 Acute phase (0–2 weeks)

Immobilization: A shoulder sling or brace is used to maintain the shoulder in internal rotation, alleviating pain and preventing further injury. Ice Therapy: Ice packs are applied for 15–20 min multiple times daily within the first 48 h post-injury to reduce swelling and pain. Analgesia: Nonsteroidal anti-inflammatory drugs (NSAIDs, e.g., ibuprofen) or acetaminophen are administered based on pain severity. Passive Mobilization: Passive range-of-motion exercises (e.g., pendulum exercises) are initiated as tolerated to prevent joint stiffness.

#### 2.4.2 Intermediate phase (2–6 weeks)

Gradual Increase in Mobility: Active-assisted range-of-motion exercises (e.g., using the contralateral arm to assist the affected side) are introduced as pain subsides. Isometric Strengthening: Isometric exercises for the rotator cuff muscles are performed to maintain muscle strength. Radiographic Assessment: x-rays are repeated every 2–4 weeks to ensure no displacement of the fracture.

#### 2.4.3 Recovery phase (6–12 weeks)

Active Mobilization: Active range-of-motion exercises are progressively increased, including flexion, abduction, internal rotation, and external rotation. Strengthening Exercises: Resistance training for the rotator cuff and deltoid muscles is initiated to restore shoulder function. Functional Training: Activities of daily living (e.g., dressing, combing hair) and light functional exercises are incorporated.

### 2.5 Surgical technique

All surgeries were performed via a standard deltopectoral approach. Fracture fixation was achieved using the Proximal Humeral Internal Locking System (PHILOS) plate (DaBo, MenXia, China), which provides angular stability through locking screws. A combination of 3.5-mm locking screws (for metaphyseal fragments) and 4.0-mm cancellous screws (for tuberosity fixation) was utilized. Additionally, high-strength sutures (#2 FiberWire®, Arthrex, Naples, FL, USA) were passed through the rotator cuff tendon insertions and secured to the plate to augment tuberosity stabilization, particularly in comminuted fractures.

### 2.6 Statistical analysis

This study employed a retrospective cohort design. Patients were stratified into two groups based on the occurrence of secondary displacement of the greater tuberosity fracture: one group without secondary displacement and another group with secondary displacement. Continuous variables (e.g., age and bone mineral density) were compared between the two groups using the Mann–Whitney *U* test, as these variables did not follow a normal distribution. Categorical variables (e.g., fracture type, dislocation status, and gender) were compared using the chi-square test or Fisher's exact test, as appropriate.

To identify independent risk factors for secondary displacement, logistic regression analysis was performed. Variables with a *p*-value < 0.05 in the univariate analysis (e.g., age, bone density, fracture type, and dislocation status) were included in the multivariate logistic regression model. Continuous variables were analyzed as continuous predictors, while categorical variables were dummy-coded. The model's goodness-of-fit was assessed using the Hosmer-Lemeshow test, and the results were reported as odds ratios (OR) with 95% confidence intervals (CI). The significance level was set at *p* < 0.05. A *post-hoc* power analysis was performed using GPower 3.1 to assess the adequacy of our sample size. With 33 secondary displacement events and an assumed odds ratio of 5.0 for key predictors (e.g., comminuted fractures), the analysis yielded a power of 89% (*α* = 0.05). This exceeds the conventional 80% threshold, confirming that our cohort was sufficiently powered to detect clinically meaningful associations.

Given the retrospective nature of the study, matching or stratification was not performed in the logistic regression analysis. Instead, potential confounding factors were controlled by including all significant variables from the univariate analysis in the multivariate model. This approach allowed for the identification of independent risk factors while minimizing bias.

## 3 Results

### 3.1 Patient baseline data

The baseline characteristics of the patients are summarized in Table 1. A total of 177 patients were included in the study, with 69 males and 108 females. The mean age was 54.61 ± 16.42 years, and the majority of fractures were simple (65.54%) rather than comminuted (34.46%). Bone mineral density analysis, based on dual-energy x-ray absorptiometry (DXA) of the lumbar spine (L1–L4), revealed that 75.71% of patients had a T-score above −2.5, while 24.29% had a T-score below −2.5 (diagnostic threshold for osteoporosis). Shoulder dislocation was present in 22.60% of patients, and 86.44% of fractures were displaced. Conservative treatment was administered to 25.42% of patients, while surgical intervention was performed in 74.58% of cases.

**Table 1:** Patient baseline data.

Variable	Mean (SD) or *n/N*
Parents	177
BMI (kg/m^2^)	25.42 ± 6.21
Mean age (years)	54.61 ± 16.42
Gender, *n* (%)	
males	69
females	108
Injured limb, *n* (%)	
left	86
right	91
Fracture type, *n*(%)	
simple fracture	116
comminuted fracture	61
Bone mineral density, *n* (%)	
T ≤2.5	43
T ≥2.5	134
Dislocation of joint, *n* (%)	
with dislocation	40
without dislocation	137
Displacement of fracture, *n* (%)	
non-displaced fracture	24
displaced fracture	153
Treatment method, *n* (%)	
Conservative treatment	45
Surgical treatment	132
Reduction quality of fracture, *n* (%)	
Good reduction	145
Poor reduction	22

### 3.2 Comparing the difference between two cohorts of patients with or without secondary displacement

The comparison of baseline characteristics between patients with and without secondary displacement is presented in Table 2. Among the 33 patients with secondary displacement, 8 (24.2%) received conservative treatment, and 25 (75.8%) underwent surgical treatment. Significant differences were observed in mean age, fracture type, bone mineral density, shoulder dislocation, and reduction quality (*p* < 0.05). However, no significant differences were found in gender, injured limb (left/right), fracture displacement, or treatment method (*p* > 0.05).

**Table 2:** Comparing the difference between two cohorts of patients with or without secondary displacement.

Variable	Non-secondary displacement	Secondary displacement	Test statistic	*P* value
Mean age (years)	51.82 ± 15.78	65.93 ± 14.45	U = 1,502.5	<0.001
Gender, *n* (%)			*χ*^2^ = 0.54	0.4640
males	55 (38.19%)	14 (42.4%)		
females	89 (61.81%)	19 (57.6%)		
Injured limb, *n* (%)			χ^2^ = 0.001	0.990
left	70 (48.61.0%)	16 (48.4%)		
right	74 (51.39%)	17 (51.5%)		
Fracture type, *n* (%)			χ^2^ = 18.32	<0.001
simple fracture	105 (72.92%)	11 (33.3%)		
comminuted fracture	39 (27.08%)	22 (66.7%)		
Bone mineral density, *n* (%)			χ^2^ = 22.45	<0.001
T ≤2.5	24 (16.67.0%)	19 (57.5%)		
T ≥2.5	120 (83.33%)	14 (42.5%)		
Dislocation of joint, *n* (%)			χ^2^ = 24.56	<0.001
with dislocation	22 (15.28%)	18 (54.5.6%)		
without dislocation	122 (84.72%)	15 (45.5%)		
Displacement of fracture, *n* (%)			χ^2^ = 3.72	0.0540
non-displaced fracture	23 (15.92%)	1 (3.0%)		
displaced fracture	121 (84.08%)	32 (7.0%)		
Treatment method, *n* (%)			χ^2^ = 0.03	0.8640
Conservative treatment	37 (25.69%)	8 (24.2%)		
Surgical treatment	107 (74.31%)	25 (75.8%)		
Reduction quality of fracture, *n* (%)			χ^2^ = 18.45	<0.001
Good reduction	126 (87.50%)	19 (57.5%)		
Poor reduction	18 (12.50%)	14 (42.5%)		

Significant statistical differences were observed between the two groups in mean age, fracture type, bone mineral density, shoulder dislocation, and reduction quality of fracture, indicating a statistically significant association (*P* < 0.05). However, no significant difference was found in gender, left/right limb**s**, displacement of fracture, and treatment method (*P* > 0.05).

### 3.3 Risk factors associated with secondary displacement of humeral greater tuberosity fractures

Logistic regression analysis revealed that comminuted fractures (OR = 8.05; 95% CI: 1.73–37.43; *p* = 0.008), osteoporosis (OR = 5.21; 95% CI: 1.10–24.73; *p* = 0.038), shoulder dislocation (OR = 29.41; 95% CI: 4.14–209.06; *p* = 0.001), and poor reduction quality (OR = 18.35; 95% CI: 1.89–178.44; *p* = 0.012) were significantly associated with secondary displacement.

## 4 Discussion

The primary aim of this study was to identify risk factors associated with secondary displacement in fractures of the humeral greater tuberosity and to provide clinical guidelines for diagnosis and treatment. Our main findings revealed that comminuted fractures, osteoporosis, shoulder dislocation, and poor reduction quality were independent risk factors for secondary displacement. These findings highlight the importance of meticulous preoperative planning and careful reduction techniques, particularly in patients with these risk factors.

The management of fractures involving the greater tuberosity of the humerus continues to pose a formidable challenge. Malunion with superior displacement of the greater tuberosity fracture can result in shoulder impingement and rotator cuff injury, ultimately compromising shoulder function (11). Although there is a lack of high-level evidence from randomized controlled trials, surgical intervention is widely accepted for cases where displacement exceeds 5 mm, a concept that has been established for over half a century (12, 13). The long-term follow-up results revealed no statistically significant difference in outcomes between patients with 0–5 mm displacement and those with 5–10 mm displacement under conservative treatment (14). The conservative management of displaced macronodular fractures larger than 5 mm has been underestimated by Sam Razaeian et al., raising doubts about the current indications for surgical intervention and necessitating further investigation with a larger patient cohort and extended follow-up periods (15). Although the standard treatment protocol for acceptable residual displacement of the greater tuberosity is widely acknowledged, it should be noted that even minor residual displacement can significantly impact shoulder biomechanics and potentially disrupt the delicate balance of forces required for arm elevation (11). The most prevalent issue that directly impacts the prognosis of the shoulder joint is the occurrence of secondary displacement of the greater tuberosity following both conservative and surgical treatments. Therefore, this study incorporated common factors that may contribute to secondary displacement of greater tuberosity fractures into a statistical analysis. The study identified fracture type, shoulder dislocation, and poor reduction as risk factors for secondary displacement of the greater tuberosity. However, other factors such as sex, age, treatment mode, and fracture displacement were not found to be significantly associated with secondary displacement of the greater tuberosity. While bone mineral density (BMD) as a continuous variable did not show a significant association, osteoporosis (defined as a T-score ≦2.5) was identified as a significant risk factor in the multivariate analysis. This suggests that patients with osteoporosis, rather than those with merely low bone mineral density, are at higher risk for secondary displacement.

In this study, unexpected findings revealed that 33 patients (18.64%) experienced secondary displacement of the greater tuberosity, indicating a higher incidence than previously anticipated and highlighting its status as a common complication. It is important to note that greater tuberosity fractures not only involve bone injury but may also encompass damage to the rotator cuff (16). While well-known classifications, such as the Neer classification, divide proximal humerus fractures into 2-part, 3-part, and 4-part fractures based on the number of displaced fragments, we chose to classify fractures as either simple (non-comminuted) or comminuted (multiple fragments) for several reasons. First, our primary focus was on identifying risk factors for secondary displacement, and we found that the distinction between simple and comminuted fractures was sufficient to capture the complexity and instability of the fractures. Comminuted fractures, regardless of the exact number of parts, consistently presented with higher instability and poorer reduction outcomes, which are critical factors in secondary displacement. Second, the Neer classification, while useful for surgical planning, is less relevant in the context of conservative management, which was also a significant part of our study population. Finally, the retrospective nature of our study limited the availability of detailed imaging data required to apply the Neer classification consistently across all cases. Future studies with larger sample sizes and more detailed imaging data may benefit from incorporating the Neer classification to provide additional insights into the relationship between fracture complexity and secondary displacement. The degree of mass disintegration of the greater tuberosity and the quality of the rotator cuff exhibit a strong correlation with the outcome of surgical repair. The findings by Liu et al. suggest that increased mass fixation of greater tuberosity fractures using proximal humeral locking system (PHILOS) plates may result in internal fixation failure and unfavorable prognosis (17). We conducted a study on 61 patients with comminuted fractures of the greater tuberosity, out of which 22 patients exhibited secondary displacement of the greater tuberosity fragments. In contrast, among 116 patients with simple fractures of the greater tuberosity, only 11 patients experienced secondary displacement. The statistical analysis revealed significant differences between the two groups in terms of fracture types (*p* < 0.05). Furthermore, logistic regression testing indicated that comminuted fractures were identified as a risk factor for secondary displacement of the greater tuberosity. These findings emphasize the importance for clinicians to engage in meticulous preoperative planning when dealing with comminuted greater tuberosity fractures. It is crucial to consider additional surgical techniques such as suture bridge or suturing during operations rather than relying solely on a single PHILOS plate.

The combination of shoulder dislocation and humerus greater tuberosity fracture accounts for approximately 5%–30% of all shoulder dislocations (18). However, the precise mechanism underlying the occurrence of humerus greater tuberosity fractures remains unclear. It is postulated that the primary injury mechanism involves impact or shear forces exerted on the anatomic neck by strong deltoid muscle contraction following acute shoulder dislocation (19). The present study reveals a potential correlation between the traumatic anterior dislocation of the shoulder joint with greater tuberosity fracture and an increase in critical shoulder angle (CSA) as well as glenoid inclination angle (GI) (20). Phob Ganokroj et al. classified greater tuberosity fractures combined with shoulder dislocation as type 3 based on x-ray analysis, and observed that greater tuberosity fractures or cumulative humeral head fractures exhibited an increased likelihood of secondary displacement following reduction (21). We observed a statistically significant disparity between the two patient groups in terms of the prevalence of greater tuberosity fractures. Logistic regression analysis indicated that shoulder dislocation was identified as a risk factor for secondary displacement of the greater tuberosity. Furthermore, our examination of cases involving large tubercle fractures combined with shoulder dislocation revealed that comminuted fractures constituted the majority among patients experiencing secondary displacement of the larger tubercles. Shoulder dislocation often causes additional soft tissue damage, including rotator cuff tears and capsular injury. These injuries can destabilize the fracture fragments, making them more prone to displacement during rehabilitation. Comminuted fractures involve multiple bone fragments, which can lead to unstable fixation and increased stress on the implant. This instability may result in fragment displacement during the early postoperative period, especially under the tension of the rotator cuff muscles. Consequently, it is imperative for clinicians to exercise heightened vigilance for potential instances of secondary displacement in greater tuberosity fractures associated with shoulder dislocation, particularly those presenting as split fractures encompassing a substantial area or involving the humeral head.

Poor reduction or residual displacement of humeral greater tuberosity fractures is a common clinical scenario. In this study, Among the 33 patients with secondary displacement, 14 (42.4%) had poor reduction quality, highlighting the significant impact of reduction quality on the risk of secondary displacement. Inadequate reduction prevents proper alignment of fracture fragments, leading to uneven stress distribution and instability. Poorly reduced fragments are more susceptible to displacement under physiological loads, particularly during early mobilization. Achieving anatomical reduction and stable fixation has always been our goal; however, it can be challenging to achieve this in every patient. A retrospective study of 98 proximal humerus fractures found that only 40 patients (40.8%) achieved an anatomically acceptable fracture reduction, and good reduction significantly reduced postoperative complications and revision rates (22). We encountered challenges in achieving anatomical or satisfactory reduction of all fractures in cases where the greater tuberosities were crushed, the internal fixation system failed to adequately cover the fracture area, or when combined with loose bones. Poorly reduced bones were found to be more susceptible to secondary displacement during postoperative rehabilitation.

Achieving a balance between proper shoulder immobilization and early rehabilitation exercise is often perceived as contradictory; however, continuous follow-up monitoring plays a pivotal role in resolving this apparent contradiction. Several studies have suggested that a 2-week period of appropriate immobilization is necessary to prevent joint adhesion, while intensive training should be postponed until full passive range of motion is achieved (23–25). It has also been suggested that continuous x-rays should be employed to ensure minimal displacement of fracture fragments following the initiation of exercise, with rehabilitative exercises being initiated after a period of 6 weeks (26). Our study revealed that secondary displacement of the tuberosity was consistently observed within the initial month following injury upon x-ray examination, suggesting a potential earlier occurrence of secondary displacement. In light of this phenomenon, we advocate for an additional follow-up assessment at approximately 1–2 weeks post-injury to evaluate the propensity for the early development of secondary displacement in the greater tuberosity.

This study has several limitations. The sample size, particularly for subgroups such as patients with shoulder dislocation and poor reduction quality, was relatively small. This may have inflated the odds ratios (ORs) for these variables, as seen in Table 3. The large ORs and wide confidence intervals suggest potential instability in the estimates, likely due to the limited number of cases. While statistically significant, these results should be interpreted cautiously, and future studies with larger sample sizes are needed for validation. The sample size of the secondary displacement group was significantly smaller than that of the non-secondary displacement group. This imbalance in sample size may have influenced the statistical results, particularly in the logistic regression analysis, where small sample sizes can lead to inflated odds ratios and wider confidence intervals. Nor did we extensively examine the criteria necessitating re-surgical intervention after secondary displacement of the greater tuberosity. Addressing these deficiencies will be a primary focus in our forthcoming research.

**Table 3:** Risk factors associated with secondary displacement of humeral greater tuberosity fractures.

Risk factors	Odds ratio (95% CI)	*P* value
Female	0.5153 (0.1561, 1.7007)	0.2764
Age ≥60	3.9932 (0.5863, 27.1977)	0.1572
Left	2.0934 (0.6826, 6.4203)	0.1963
Comminuted fracture	11.6139 (2.7801, 48.5183)	0.0008
Osteoporosis	5.2066 (1.0962, 24.7295)	0.0379
Dislocation of shoulder joint	43.7907 (6.5343, 293.4706)	0.0001
Displacement of fracture	0.4861 (0.0279, 8.4633)	0.6207
Surgical treatment	3.1455 (0.4506, 21.9575)	0.2477
Poor reduction of fracture	33.1242 (3.9479, 277.9225)	0.0013

Logistic regression analysis revealed that the presence of comminuted fracture of the greater tuberosity of humerus, osteoporosis, dislocation of the shoulder joint, and poor reduction of fracture were identified as significant risk factors for secondary displacement of the greater tuberosity of humerus.

## 5 Conclusions

Comminuted fractures of the greater tuberosity, osteoporosis (defined as a T-score ≦2.5), shoulder dislocation, and poor reduction have been identified as risk factors for secondary displacement of greater tuberosity fractures. While bone mineral density as a continuous variable was not significantly associated with secondary displacement, the presence of osteoporosis was a significant risk factor. During clinical diagnosis and treatment, it is crucial to acknowledge the adverse prognosis associated with these conditions and to ensure comprehensive preoperative planning, along with fulfilling detailed notification obligations.

## Data Availability

The original contributions presented in the study are included in the article/Supplementary Material, further inquiries can be directed to the corresponding authors.
